# Polyvinylpyrrolidone-Based Bio-Ink Improves Cell Viability and Homogeneity during Drop-On-Demand Printing

**DOI:** 10.3390/ma10020190

**Published:** 2017-02-16

**Authors:** Wei Long Ng, Wai Yee Yeong, May Win Naing

**Affiliations:** 1 Singapore Centre for 3D Printing (SC3DP), School of Mechanical and Aerospace Engineering, Nanyang Technological University (NTU), 50 Nanyang Avenue, Singapore 639798, Singapore; ng0138ng@e.ntu.edu.sg; 2 Singapore Institute of Manufacturing Technology (SIMTech), Agency for Science, Technology and Research, 73 Nanyang Drive, Singapore 637662, Singapore; winnaingm@simtech.a-star.edu.sg

**Keywords:** bioprinting, bio-inks, drop-on-demand, 3D printing, additive manufacturing

## Abstract

Drop-on-demand (DOD) bioprinting has attracted huge attention for numerous biological applications due to its precise control over material volume and deposition pattern in a contactless printing approach. 3D bioprinting is still an emerging field and more work is required to improve the viability and homogeneity of printed cells during the printing process. Here, a general purpose bio-ink was developed using polyvinylpyrrolidone (PVP) macromolecules. Different PVP-based bio-inks (0%–3% w/v) were prepared and evaluated for their printability; the short-term and long-term viability of the printed cells were first investigated. The Z value of a bio-ink determines its printability; it is the inverse of the Ohnesorge number (Oh), which is the ratio between the Reynolds number and a square root of the Weber number, and is independent of the bio-ink velocity. The viability of printed cells is dependent on the Z values of the bio-inks; the results indicated that the cells can be printed without any significant impairment using a bio-ink with a threshold Z value of ≤9.30 (2% and 2.5% w/v). Next, the cell output was evaluated over a period of 30 min. The results indicated that PVP molecules mitigate the cell adhesion and sedimentation during the printing process; the 2.5% w/v PVP bio-ink demonstrated the most consistent cell output over a period of 30 min. Hence, PVP macromolecules can play a critical role in improving the cell viability and homogeneity during the bioprinting process.

## 1. Introduction

The ability to control spatial positioning of multiple types of cells in an accurate and repeatable manner is of great interest to a variety of biological applications including multiplexed cell microarrays [1], in-vitro drug screening [2], and even tissue engineering [3]. Recently, 3D bioprinting technology has emerged as a leading platform for directed cell deposition [4,5] which includes laser-assisted [6,7,8], extrusion-based [9,10,11,12,13,14,15,16], microvalve-based [17,18,19,20], and inkjet-based [21,22,23] bioprinting systems. Particularly, the drop-on-demand (DOD) bioprinting approach has several advantages such as its delicate control over the deposition pattern and material volume at pre-defined positions that facilitate precise deposition of different types of cells and biomaterials without contact with the underlying substrates [24]. DOD printing is traditionally used for the deposition of non-biological materials such as polymers [25] or inorganic inks [26] and translation of this technology into cell-based bioprinting is still a relatively new field [27]. 

To date, numerous studies have been conducted to evaluate the effects of printing pressure [28,29,30], nozzle diameter [28,29,30], and substrate stiffness [31] on cell viability during the printing process. Furthermore, another challenging issue faced in most bioprinting systems is the cell sedimentation effect, which occurs as a result of gravitational forces and changes the cell homogeneity within the printing cartridge over time. This cell sedimentation effect on bioprinter output was characterized [32], highlighting the issue of inconsistent printing output over time. Some attempts to mitigate the sedimentation effects include the use of ethylene diamine tetra-acetic acid (EDTA) [33] and neutral buoyancy [34]. However, the use of EDTA can be detrimental to cell viability and the direct measurement of cell density poses a huge obstacle due to the lack of sophisticated measuring equipment (most measurements are only limited to indirect means through density centrifugation or optical techniques) [34]. As such, the key limitation that hindered the prevalent use of cell-based bioprinting is due to the poor viability and homogeneity of the printed cells [35].

In this paper, we developed a general purpose bio-ink using polyvinylpyrrolidone (PVP) that improves the viability of the printed cell and mitigates the cell adhesion and sedimentation during the printing process. A systematic investigation was performed to analyze the influence of the polyvinylpyrrolidone on both the viability and homogeneity of printed cells using a commercially-available bioprinter (RegenHu, Biofactory).

## 2. Materials and Methods

### 2.1. Cell Culture

Neonatal human foreskin fibroblasts (HFF-1 from ATCC^®^ SCRC-1041™, Virginia, WV, USA) were used in this study. The cells were cultured in complete growth medium that comprises high glucose DMEM with l-glutamine, supplemented with 15% FBS (fetal calf serum). The culture medium was changed every 3 days and the cells were routinely passaged in tissue culture flasks (Passages 3–5). The adherent HFF-1 cells were harvested using 0.25% trypsin/ethylene diamine tetra-acetic acid (EDTA) at 90% confluency.

### 2.2. Bio-Ink Synthesis

We prepared different types of PVP-based bio-inks (0%–3% w/v PVP), which were used in all experiments. The dimensionless Z value is the inverse of the Ohnesorge number (Oh), which is defined as the ratio between the Reynolds number and the square root of the Weber number, and is independent of the bio-ink velocity. To evaluate the influence of the polymer concentration on the Z values, different concentrations of polyvinvylpyyrolidone (MW: 360 kDa) were added to complete growth medium (as mentioned earlier) containing 1 mil HFF-1 cells/mL to formulate different PVP-based bio-inks (0%–3% w/v PVP). To evaluate the influence of cell concentration on the Z values, we added different cell concentrations (0.5–2.5 mil cells/mL) to 2.5% w/v PVP-based bio-ink.

### 2.3. Bio-Ink Characterization

To investigate the influence of polymer concentration on the properties (Z values) of the PVP-based bio-inks, different concentrations of PVP polymer (MW: 360 kDa) and a constant cell concentration of one million cells/mL were added to the complete growth medium (DMEM supplemented with 15% fetal bovine serum) to formulate different PVP-based bio-inks (0%–3% w/v). Measurements were then performed on the different PVP-based bio-inks to evaluate the effect of varying polymer concentration (0%–3% w/v) on viscosity, surface tension, and density of the different PVP-based bio-inks and their respective Z values. The rheological properties of the PVP-based bio-inks were evaluated using the Discovery hybrid rheometer (TA instruments, New Castle, DE, USA). The values of the strain amplitude were first verified to ensure that all measurements were performed within the linear viscoelastic region. Next, the viscosities of different PVP-based bio-inks were evaluated for shear rates ranging from 0.1 to 1000 s^−1^ at a constant temperature of 27 °C (similar temperature as our printer platform). The surface tension of the bio-inks was measured using the capillary rise method (reference to water), and a weighing balance was used to measure the density of the bio-inks (weight per ml of bio-ink). All measurements were conducted in triplicate.

### 2.4. Drop-On-Demand Printing of Cell Droplets

All experiments were conducted using a 3-D microvalve-based bioprinter (RegenHU Biofactory^®^, Fribour, Switzerland). We sterilized the microvalve-based print-heads using 70% ethanol and performed 30 min of UV sterilization prior to printing. The PVP-based bio-inks were loaded into a sterile printing cartridge (5 mL), which was subsequently attached to a microvalve-based printhead with a 100 µm nozzle diameter. A constant printing pressure of 0.25 bar was applied throughout all of the experiments; 15 arrays of 3 × 3 cell droplets (*n* = 135) were printed onto the Corning^®^ tissue-culture treated culture dishes (35 mm × 10 mm) and evaluated for its short-term and long-term viability using the Molecular Probes^®^ Live/Dead staining kits (Life-Technologies, Eugene, OR, USA) and PrestoBlue^®^ assay (Frederick, MD, USA), respectively. For the short-term viability test, the live/dead staining assay was loaded in a different print-head and printed directly over the printed cell droplets. For the long-term viability test, culture medium was added to the samples immediately after printing and the samples were kept in an incubator at 37 °C in 5% CO_2_ for up to 96-h to evaluate the influence of Z values on the long-term viability of the printed cells. 

### 2.5. Statistical Analysis 

All experimental results are presented as mean ± standard deviation. Statistical comparisons were performed using Student’s *t*-test. Significance levels were as follows: *p* < 0.005 (***), *p* < 0.05 (*). Values were considered to be significantly different when the *p* value was <0.05.

## 3. Results and Discussion

### 3.1. Influence of Polymer Concentration and Cell Concentration on the Short-Term Viability of Printed Cells

The three key properties (viscosity, surface tension, and density) of bio-inks influence the printability; an approximate solution to the Navier-Stokes equations for printability of the bio-inks can be represented by the Reynolds number (*N_Re_*: the ratio of inertial to viscous forces) and the Weber number (*N_We_*: a balance between the inertial and capillary forces). The dimensionless Z value is the inverse of the Ohnesorge number (Oh), which is defined as the ratio between the Reynolds number and the square root of the Weber number, and is independent of the bio-ink velocity.

(1) $$N_{Re}=\frac{vr\rho }{\eta }$$

(2) $$N_{We}=\frac{v^{2}r\rho }{\gamma }$$

(3) $$Z=\frac{N_{Re}}{{\left(N_{We}\right)}^{1/2}}=\frac{{\left(r\rho \gamma \right)}^{1/2}}{\eta }$$

where v, ρ, η, and γ are the average travel velocity, density, viscosity, and surface tension of the bio-inks, respectively, and r is a characteristic dimension (radius of the nozzle orifice).

Different concentrations of PVP polymer and a constant cell concentration of 1 million cells/mL were added to the complete growth medium (DMEM supplemented with 15% fetal bovine serum) to formulate different PVP-based bio-inks (0%–3% w/v). From the measurements, both the viscosity and density of the PVP-based bio-inks increase with increasing PVP concentration, whereas the surface tension of PVP-based bio-inks decreases with increasing PVP concentration. Overall, this results in a lower Z value with increasing PVP concentration (from a Z value of 64.36 in 0% w/v PVP-based bio-ink to a Z value of 3.73 in 3% w/v PVP-based bio-ink, as shown in Table 1).

**Figure 1:**
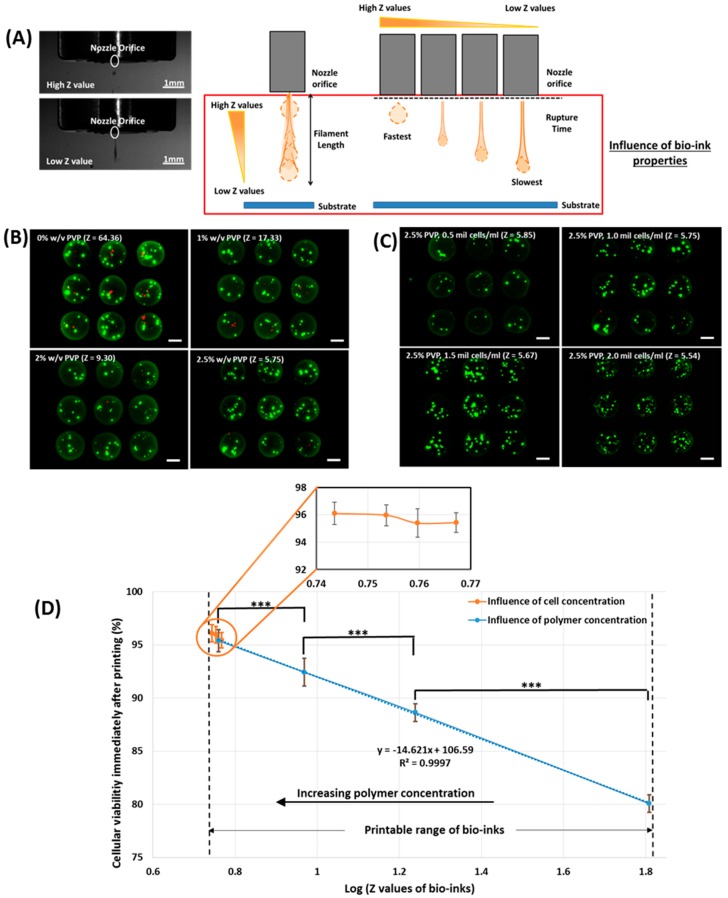
(**A**) (**Left**) Representative images of droplet generation at both low and high Z values (**Right**) Schematic drawing of the printing process; (**B**) Influence of polymer concentration (0%–2.5% w/v, constant cell concentration of 1 mil cells/mL) on Z values and corresponding viability; (**C**) Influence of cell concentration (0.5–2 mil cells/mL, constant polymer concentration of 2.5% w/v) on Z values and corresponding viability (scale bar: 200 µm); (**D**) Short-term viability of printed cell droplets (*n* = 135) immediately after printing (mean ± SD). Significance levels are as follows: *p* < 0.005 (***), *p* < 0.05 (*).

A constant printing pressure of 0.25 bar was applied for all the PVP-based bio-inks; as it was previously demonstrated that the detrimental effect of shear stress was observed when the printing pressure is more than 0.25 bar [30]. The printable range of Z values for the PVP-based bio-inks was determined to be within 5.75 ≤ Z ≤ 64.36 (0%–2.5% w/v); the 3% w/v PVP-based bio-ink with a Z value of 3.73 shows poor printability as the lower limit of Z is governed by the maximum printable viscosity of the bio-ink [36]. It was observed that the short-term viability of printed cells (immediately after printing) increases with decreasing Z values (from 80.1% in 0% w/v PVP, Z = 64.36% to 95.4% in 2.5% w/v PVP, Z = 5.75); a bio-ink with a lower Z value generally has a lower droplet velocity due to the slow filament elongation and long rupture time [36] and the higher viscosity provides an additional cushioning effect (higher energy dissipation) for the printed cells [37,38] (Figure 1).

Further investigation was then conducted to evaluate the influence of cell concentration on Z values and their corresponding short-term viability of printed cells. The 2.5% w/v PVP-based bio-ink was selected (highest short-term viability among all the PVP-based bio-inks) and varying cell concentration (0.5–2.5 mil cells/mL) was added to the polymer solution. It was observed that the Z value decreases with increasing cell concentration (Table 1). The viscosity of the bio-ink increases with increasing cell concentration due to the distortion of fluid flow and frictional forces at the cell-fluid interface. Furthermore, it also reduces the surface tension due to a reduction in total free energy of the bio-ink (more cells are adsorbed to the interface). A similar study has corroborated our experimental data [39] an increase in cell concentration (from 0.5 mil cells/mL to 2.5 mil cells/mL) only results in a slightly lower Z value (from 5.85 to 5.38). No significant difference in the short-term viability of printed cells was observed when the cell concentration increased from 0.5 mil cells/mL (Z = 5.85) to 2.0 mil cells/mL (Z = 5.54) (poor printability was observed when the cell concentration increased beyond 2.0 mil cells/mL).

Interestingly, the short-term viability of the printed cells follows a linear relationship with respect to the log Z values within the printable range of Z values (5.54 ≤ Z ≤ 64.36) and the short-term viability of the printed cells generally increases with decreasing Z values (Figure 1). This highlights that the properties of the bio-inks (Z values) have significant effects on the short-term viability of the printed cells, indicating that a printable bio-ink with a low Z value (Z < 9.3) is ideal for maintaining high cell survival rates (>90%).

### 3.2. Influence of Polymer Concentration on the Long-Term Viability of Printed Cells

**Figure 2:**
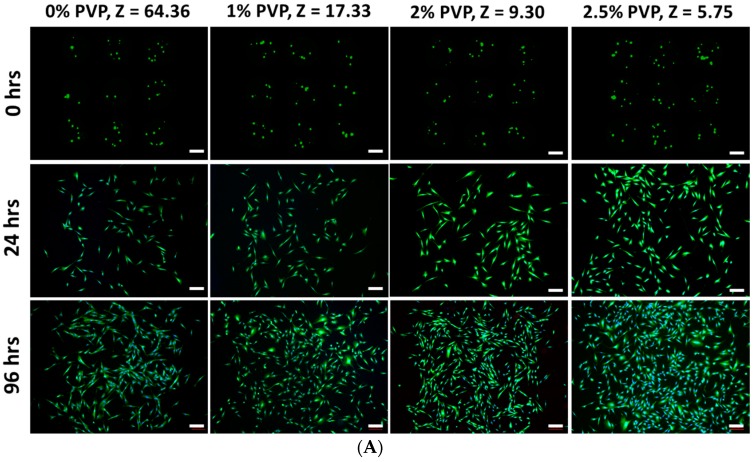

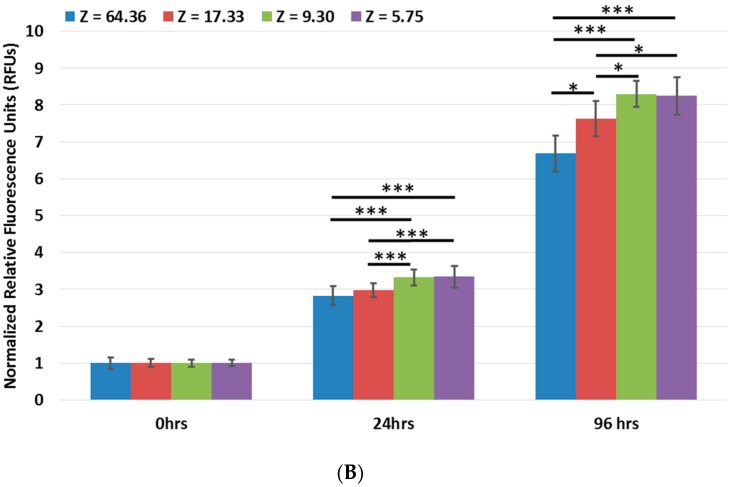
(**A**) Representative images of printed cells (constant cellular concentration of 1.0 mil cells/mL) at different time intervals (scale bar: 200 µm); (**B**) A graph showing the long-term viability of printed cell droplets at different time intervals post-printing (mean ± SD). Significance levels are as follows: *p* < 0.005 (***), *p* < 0.05 (*).

Further study was conducted to monitor the influence of bio-ink properties (Z values) on the long-term viability of the remaining viable fibroblast cells (up to 96 h) that survived the printing process at 0-, 24-, and 96-h post-printing using a quantitative cell proliferation assay (Figure 2). The number of cells at any given time point is expressed in relative fluorescence units (RFUs). As discussed in the earlier section, the Z value of the bio-inks influences the short-term viability of the printed cells (more viable cells in PVP-based bio-inks with lower Z values). As such, normalization of all the RFUs data (0 to 96 h) was performed with respect to the 0-h of each respective PVP bio-ink (Z = 64.36, 17.33, 9.30, and 5.75) to ensure a fair comparison for long-term viability of the printed cells among all the different PVP-based bio-inks. It was found that the PVP-based bio-inks with Z values ≤9.30 (2% and 2.5% w/v) have significantly higher normalized RFUs than both 0% and 1% w/v PVP-based bio-inks (Z = 64.36 and Z = 17.33, respectively) at both 24- and 96-h intervals. Although there was no significant difference between the normalized RFUs of the 0% w/v PVP-based bio-ink (Z = 64.36) and the 1% w/v PVP-based bio-ink (Z = 17.33) at the 24-h interval, a significantly higher normalized RFU in the 1% w/v PVP-based bio-ink (Z = 17.33) compared to the 0% w/v PVP-based bio-ink (Z = 64.36) was observed at the 96-h interval. Furthermore, there was no significant difference between the normalized RFUs of 2% w/v PVP-based bio-ink (Z = 9.30) and 2.5% w/v PVP-based bio-ink (Z = 5.75) at both the 24- and 96-h intervals, which indicated that the printed cells in bio-inks with Z values (≤9.30) did not suffer from either short-term or long-term printing-induced damage. The findings demonstrated that the Z values of the bio-ink not only influence the short-term cell viability but also induce long-term alterations in the proliferation profile of the printed cells that survived the bioprinting process. These findings indicated that below a specific Z value threshold of ≤9.30 (2%, 2.5% w/v PVP), the cells can be printed without any short-term or long-term impairment.

### 3.3. Influence of Polymer Concentration on the Homogeneity of Printed Cells 

**Figure 3:**
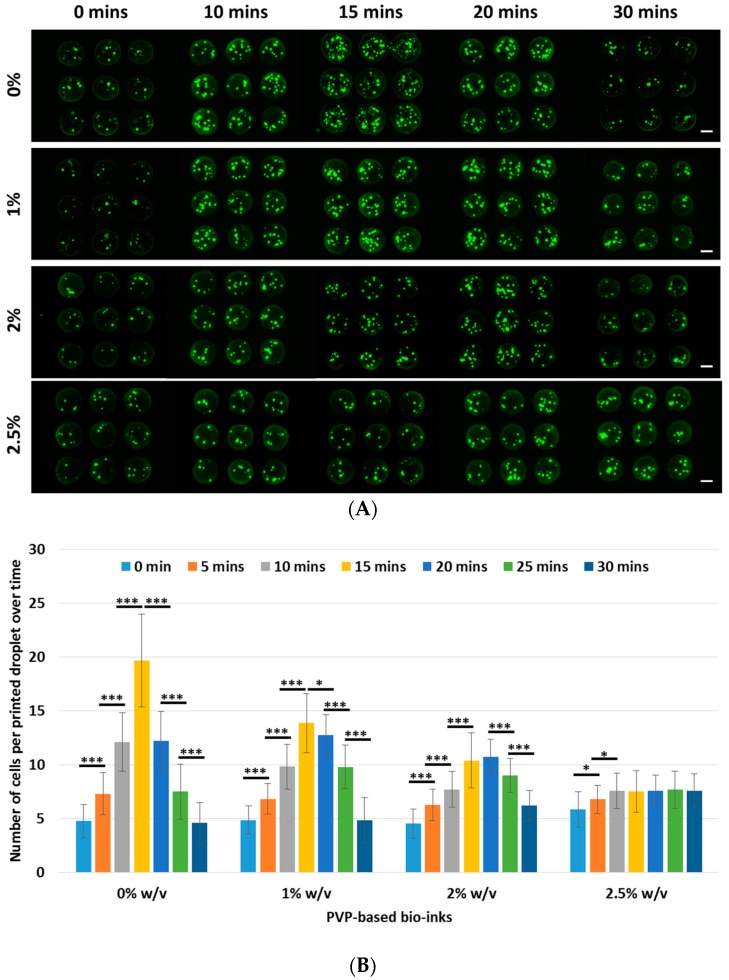
(**A**) Representative images of printed cells (constant cellular concentration of 1.0 mil cells/mL) at different time intervals (scale bar: 200 µm); (**B**) A graph showing the number of cells per printed droplet over time (mean ± SD). Significance levels are as follows: *p* < 0.005 (***), *p* < 0.05 (*).

A 30-min printing window is considered reasonable as a long printing process window may have adverse effects on the cell viability [40,41]. The number of cells per printed droplet across all the groups (0%–2.5% w/v) was relatively constant at the 0-min interval. The initial increase in cell output across all the groups (from 0- to 15-min interval) occurred mainly as a result of cell sedimentation; an increasing PVP concentration mitigated the sedimentation effect and the most pronounced sedimentation effect was observed in the 0% w/v PVP-based bio-inks. Generally, the number of cells per printed droplet increased until a maximum cell output before it started to reduce over time for all the groups (0%–2% w/v PVP-based bio-inks), as shown in Figure 3. In contrast, the use of the 2.5% w/v PVP-based bio-ink resulted in an initial increase in cell output before it reached a steady-state concentration.

The underlying phenomena that influence the cell homogeneity within the printing cartridge over time have yet to be fully understood. A mathematical model was developed to predict the cell concentration in the printing cartridge over time [32]; the cell output is expected to increase linearly over time until it reaches a constant steady-state output. However, it was reported that the experimental results differed significantly from the cell output model. There is a gradual initial increase in cell output (sedimentation effect), followed by a decrease in the cell output (suggesting the presence of some other phenomenon). As the experiment did not fully utilize the approximately 1,000,000 cells (1 mil cells/mL) in the printing cartridge, the decrease in cell output was clearly not a result of cell depletion in the printing cartridge.

**Figure 4:**
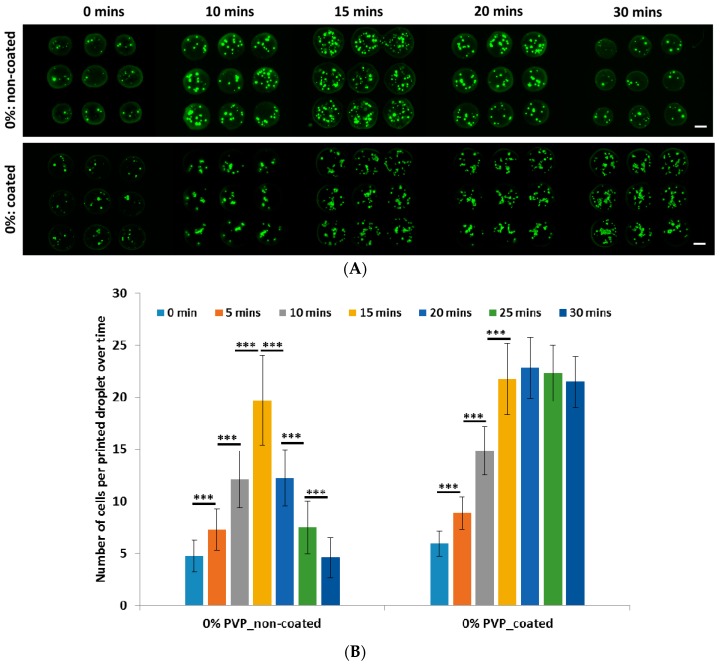
(**A**) Representative images of printed cells in non-coated and coated printing cartridges (constant cellular concentration of 1.0 mil cells/mL) at different time intervals (scale bar: 200 µm); (**B**) A graph showing the number of cells per printed droplet over time (mean ± SD). Significance levels are as follows: *p* < 0.005 (***), *p* < 0.05 (*).

Some earlier works reported that the van der Waals interactions between the cells and the interior surface of the printing cartridge [42,43] esulted in cell adhesion along the constriction of the printing cartridges. The free-floating cells were subsequently attracted to the adhered cells and contributed to the growth of cell aggregates near the constriction at the bottom of the printing cartridge. We hypothesized that the presence of PVP mitigates the cell sedimentation effect and also reduces cell adhesion within the printing cartridge during the printing process. To test our hypothesis (presence of PVP polymer prevents cell adhesion), we performed a pre-coating of the 2.5% w/v PVP solution within the printing cartridges overnight at 4 °C. Next, we evaluated the cell output for the 0% w/v PVP-based bio-ink (coated vs. non-coated) over a period of 30 min. It was observed that pre-coating of the PVP solution in the printing cartridge improved the cell output for the 0% w/v PVP-based bio-inks over time (as compared to the non-coated groups); the cell output increased linearly (from 0 to 15 min) until it reached a constant steady-state output (beyond 15 min), as shown in Figure 4. This indicated that the presence of PVP polymer prevents cell adhesion [44] and improves the cell output over time. A comparison between the non-coated and pre-coated groups clearly indicated that the cell adhesion effect became more pronounced over time, resulting in a significantly lower cell output with longer printing times. 

The use of PVP-based bio-inks helped to mitigate cell adhesion and sedimentation during the printing process and improved the cell output consistency over time.

## 4. Conclusions

Bioprinting is a highly complex deposition process; the printed cells first experienced high shear stress in the printing nozzle, followed by the droplet impact after being ejected from the nozzle orifice. The addition of PVP molecules alters the Z value, which influences the printability of the bio-inks (5.54 ≤ Z ≤ 64.36). It results in lower droplet velocity due to the slow filament elongation and long rupture time and also provides an additional cushioning effect (higher energy dissipation) for the printed cells upon landing on the substrate surface, hence improving the viability of the printed cells during the DOD printing process. Within this printable range of Z values, a decreasing Z value generally results in higher shorter-term cell viability (inversely proportional to log Z values). A change in cell concentration (0.5 mil cells/mL to 2.0 mil cells/mL) has no significant effect on the short-term cell viability due to the negligible change in log Z values (Figure 1). Furthermore, cells can be printed without any significant short-term (immediately after printing) or long-term (up to 96-h) impairments using bio-inks within a specific Z value threshold of Z ≤ 9.30. Even though the presented data are specific for microvalve-based bioprinting processes, this can also be applicable to other nozzle-based DOD bioprinting systems (whereby the printability of the bio-inks is governed by the Z values). This work pioneers the investigation of Z values on the viability of printed cells (up to 96-h) during DOD bioprinting processes; this provides critical information for the formulation of new printable bio-inks that facilitates the deposition of highly viable cells. Furthermore, the presence of PVP macromolecules also mitigates the cell adhesion and sedimentation inside the printing cartridge during the printing process. Our results indicated that the PVP-based bio-inks improved the viability and homogeneity of the printed cells.

## Figures and Tables

**Table 1:** Influence of polymer concentration and cell concentration on properties (Z values) of PVP-based bio-inks and their corresponding short-term cell viability.

PVP Concentration (w/v)	Cell Concentration (mil Cells/mL)	Density (kg/m^3^)	Viscosity (mPa·s)	Surface Tension (mN/m)	Nozzle Radius (µm)	Z	Short-Term Viability (%)
0%	1.0	1001.3 ± 3.9	0.85 ± 0.05	59.8 ± 0.2	50	64.36	80.1 ± 0.83
1%	1.0	1009.3 ± 2.8	2.94 ± 0.03	51.5 ± 0.2	50	17.33	88.6 ± 0.83
2%	1.0	1020.3 ± 2.7	5.29 ± 0.05	47.5 ± 0.2	50	9.30	92.4 ± 1.30
2.5%	0.5	1024.3 ± 2.3	8.08 ± 0.13	43.7 ± 0.2	50	5.85	95.4 ± 0.71
2.5%	1.0	1025.3 ± 3.1	8.19 ± 0.14	43.2 ± 0.2	50	5.75	95.4 ± 1.04
2.5%	1.5	1026.3 ± 2.4	8.26 ± 0.13	42.7 ± 0.2	50	5.67	95.9 ± 0.78
2.5%	2.0	1026.2 ± 2.8	8.41 ± 0.14	42.3 ± 0.2	50	5.54	96.1 ± 0.82
2.5%	2.5	1027.2 ± 2.7	8.65 ± 0.15	42.1 ± 0.2	50	5.38	-
3%	1.0	1029.8 ± 3.2	12.43 ± 0.20	41.7 ± 0.2	50	3.73	-
